# Neonatal mpox in Pakistan: a case study in infection prevention and control gaps and opportunities for improvement

**DOI:** 10.1016/j.ijregi.2026.101028

**Published:** 2026-09-25

**Authors:** Mariyam Sarfraz, Nadia Noreen, Nasim Akhtar

**Affiliations:** 1 Health Services Academy, Islamabad, Pakistan; 2 Border Health Services, Islamabad, Pakistan; 3 Ministry of National Health Services, Regulations and Coordination, Khosar Block, Islamabad, Pakistan; 4 Pakistan Institute of Medical Sciences, Islamabad, Pakistan

**Keywords:** Mpox, Clade Ib, Vertical transmission, Pediatric mortality, Infection prevention and control, International Health Regulations (2005)

## Abstract

**Background:**

In 2026, a cluster of neonatal deaths from mpox clade Ib was identified in Khairpur, Sindh, marking Pakistan’s first pediatric mpox mortality and its first detection of clade Ib. The country had previously recorded only imported clade IIb cases. The cluster mirrors earlier iatrogenic pediatric human immunodeficiency virus (HIV) outbreaks in Ratodero, Mirpur Khas, and Taunsa, and recurrent nosocomial Crimean-Congo hemorrhagic fever (CCHF) outbreaks.

**Methods:**

A structured comparative clinical and case review reports analysis of six infectious disease outbreaks in Pakistan (2019-2026) was conducted. Peer-reviewed literature was identified through PubMed, Scopus, and Google Scholar (January 2021-September 2026) using outbreak-specific terms combined with “Pakistan,” “iatrogenic,” “nosocomial,” “infection prevention and control,” and “International Health Regulations (IHR).” Gray literature was retrieved from World Health Organization (WHO) country and joint-mission reports, Pakistan’s National Institutes of Health advisories, provincial acquired immunodeficiency syndrome control program dashboards, ProMED-mail, and named journalistic investigations where peer-reviewed evidence was not yet available. Outbreaks were compared against IHR 2005 core capacities and the tripartite One Health framework. Infection prevention and control (IPC) failures were assessed by cross-referencing outbreak reports with WHO minimum IPC requirements and the 2025 IPC Assessment Framework assessments in Sindh.

**Results:**

Across the six outbreaks, more than 2050 children were affected between 2019 and 2026. The Khairpur cluster (26 confirmed cases, 50% infants under six months, eight neonates) is consistent with maternal-infant vertical transmission and healthcare-associated amplification, not with adult sexual networks. A separate Karachi cluster (n = 8) demonstrated sustained human-to-human transmission from an index case with recent travel to Central Africa. Points-of-entry syndromic screening missed clade Ib because concealed lesions evade visual assessment. Cross-cutting failures include syringe reuse by unregulated practitioners, driving pediatric HIV; personal protective equipment and isolation shortfalls causing fatal nosocomial CCHF among staff each Eid-ul-Azha; and absent integrated One Health surveillance.

**Conclusion:**

The 2026 neonatal deaths reflect unreformed clinical practice, not a novel pathogen. Priorities include enforceable provincial IPC audits, a ban on unregulated practitioners, molecular-epidemiology-linked points-of-entry surveillance, a clinical pathway for mpox in pregnancy and in neonates, regional-language risk communication, and Modified Vaccinia Ankara - Bavarian Nordic for Mpox and antiviral stockpiling.

## Highlights

• First neonatal mpox clade Ib cluster reported in Khairpur, Sindh, Pakistan (2026).
• Cluster mirrors iatrogenic human immunodeficiency virus outbreaks: Ratodero, Mirpur Khas, Taunsa, Karachi.
• Same Infection prevention and control gaps drive recurrent nosocomial Crimean-Congo hemorrhagic fever outbreaks each Eid-ul-Azha.
• Points-of-entry surveillance built for clade IIb misses clade Ib routes.
• Pakistan needs a clinical pathway for mpox in pregnancy and neonatal care.

## Graphical abstract

**Figure:**
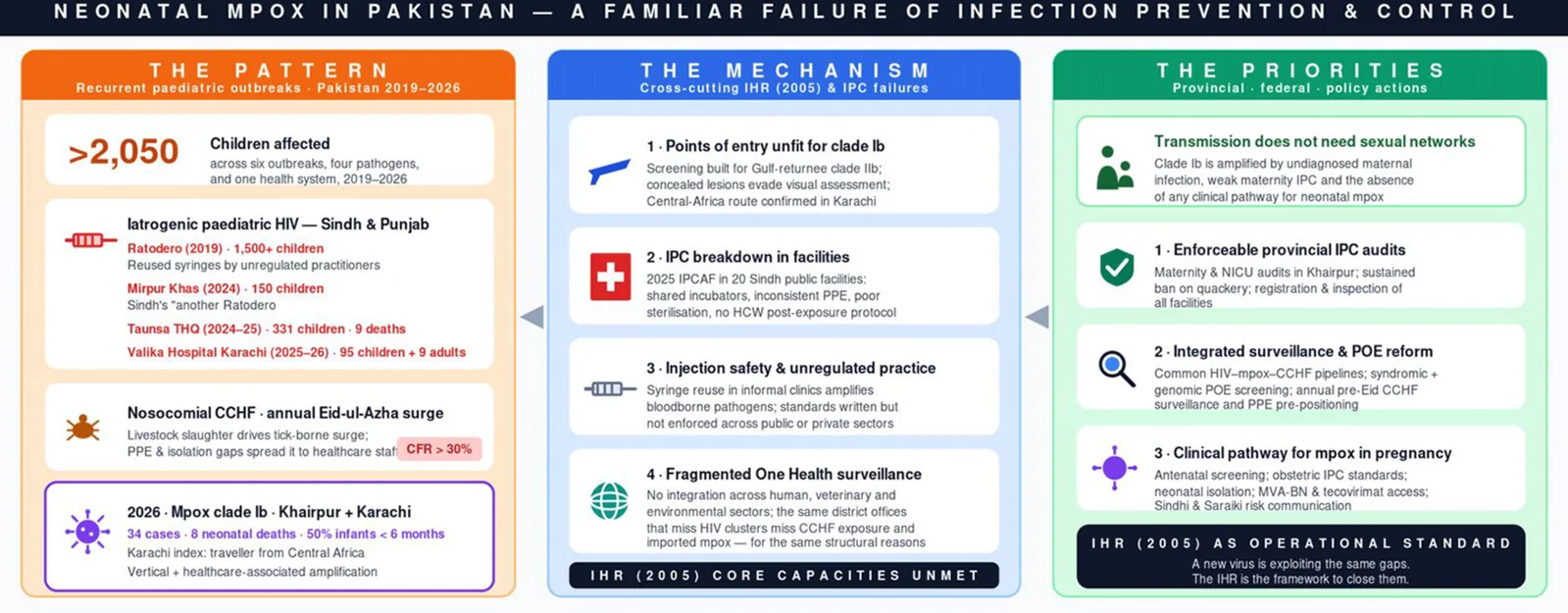

## Introduction

Across Sindh between January and April 2026, 34 Mpox cases were confirmed; 26 in Khairpur and Sukkur districts and a separate Karachi cluster (08) with documented sustained human to human transmission from a Central African travel associated index case. The cluster, identified in 2026, marks the first significant pediatric mortality from the monkeypox virus in Pakistan since the country’s first imported case in April 2023, which belonged to African clade IIb, lineage A.2.1 [1,2]. Mpox is transmitted through close contact with skin lesions, body fluids, respiratory droplets, or contaminated surfaces. Immunocompromised individuals, including those with advanced human immunodeficiency virus (HIV) disease, pregnant women, and children under 5 years, are at a higher risk of severe disease. Clade I infection is more severe than clade II, and transplacental transmission to the fetus can result in stillbirth; both vertical transmission and close contact with an infected mother after birth are established routes [1,3]. The Khairpu-Sukkur pattern is consistent with maternal-infant vertical transmission and healthcare associated amplification, not adult sexual networks.

While most globally reported cases have been associated with adult transmission, emerging clusters among neonates represent a concerning epidemiological shift. Pooled prospective cohort data from the Democratic Republic of the Congo (DRC) demonstrate that maternal and neonatal outcomes from clade I infection during pregnancy are severe, with fetal loss reported in half or more of the pregnancies in some series [3,4]. The Khairpur outbreak is also the latest in a sequence of Pakistani outbreaks in which weak infection prevention and control (IPC) has done most of the damage. In 2019, more than 1500 children in Ratodero, Sindh, tested positive for HIV after exposure to reused syringes by unregulated practitioners, with case-control work confirming iatrogenic transmission as the predominant mode [5,6]. In 2024, Mirpur Khas district in Sindh recorded 150 pediatric HIV diagnoses, with health authorities describing the district as another Ratodero [7]. Between November 2024 and October 2025, 331 pediatric HIV infections were linked to repeated syringe reuse at Tehsil Headquarters Hospital Taunsa, Punjab, with nine deaths; this pattern is now recognized in the peer-reviewed literature as a healthcare-acquired crisis [[8], [9], [10]]. Most recently, an outbreak at Kulsoom Bai Valika Social Security Hospital in SITE Town, Karachi (first reported by the World Health Organization [WHO] in November 2025) had, by early 2026, produced 95 confirmed pediatric and nine adult HIV/acquired immunodeficiency syndrome (AIDS) cases (Ministry of National Health Services, Regulations and Coordination, personal communication, January 2026); parents of affected children publicly attributed transmission to repeated reuse of 10 mL disposable syringes by nursing staff, and 40,000-50,000 HIV screening test kits have since been deployed for community contact screening [11].

Crimean-Congo hemorrhagic fever (CCHF) provides the same lesson at an annual frequency. CCHF is endemic in Pakistan, with case numbers rising each year around Eid-ul-Azha because livestock slaughter increases tick exposure and contact with infected blood [12]. Hospital admissions of severe cases have repeatedly produced nosocomial transmission, including a tertiary-care outbreak documented in 2024, in which most secondary cases were healthcare workers [13]. The case-fatality rate in nosocomial CCHF reaches 30% and above. The same gaps in personal protective equipment (PPE) supply, isolation capacity, and IPC training that allow pediatric HIV outbreaks in district hospitals allow viral hemorrhagic fever to spread within tertiary centers. The annual cycle of Eid-ul-Azha makes CCHF the most predictable IPC test Pakistan faces, and it is the one most consistently failed.

These outbreaks involve different pathogens, different routes, and different victims, but they share a mechanism: unsafe clinical practice in a health system that does not enforce its own standards.

## Methods

This is a structured comparative analysis of six infectious disease outbreaks in Pakistan (2019-2026), synthesizing primary outbreak reports, clinical and case review reports, peer-reviewed literature, and national and international surveillance documents to identify common IPC and International Health Regulations (IHR 2005) core capacity failures. The six outbreaks comprise: four iatrogenic paediatric HIV outbreaks (Ratodero 2019; Mirpur Khas 2024; Taunsa 2024–25; Kulsoom Bai Valika Social Security Hospital, SITE Town, Karachi 2025–26); the 2026 Sindh mpox clade Ib outbreak, which the WHO joint mission reports as a single provincial outbreak with two foci (Khairpur-Sukkur and Karachi) [14]; and recurrent nosocomial Crimean-Congo haemorrhagic fever, exemplified by the 2024 tertiary-care outbreak [13].

*Search strategy:* PubMed, Scopus, and Google Scholar were searched for articles published between January 2021 and September 2026 using the terms (“mpox” OR “monkeypox”) AND “Pakistan”; (“HIV” AND “Pakistan” AND (“children” OR “outbreak” OR “iatrogenic”)); (“Crimean-Congo haemorrhagic fever” OR “CCHF”) AND “Pakistan” AND (“nosocomial” OR “healthcare worker”); (“International Health Regulations” OR “IHR 2005”) AND “Pakistan”; and (“infection prevention and control” OR “IPC”) AND “Pakistan.” Titles and abstracts in English were screened; primary outbreak reports, case-control studies, national surveillance briefs, and WHO country and joint-mission reports were retained. Gray literature was identified from WHO, Pakistan’s National Institutes of Health, the Sindh AIDS Control Programme, the Punjab Primary and Secondary Healthcare Department, and ProMED-mail.

*Data sources:* for each outbreak, case counts, age distribution, transmission route, timing, geographical setting, and documented IPC and surveillance gaps were extracted. Primary sources included the WHO joint-mission preliminary report on the 2026 Sindh mpox outbreak [14]; peer-reviewed publications on the 2019 Ratodero HIV outbreak [5,6]; a *Lancet HIV* 2026 analysis of pediatric HIV outbreaks in Pakistan covering Taunsa and Mirpur Khas [8]; a 2024 *Journal of Hospital Infection* report of nosocomial CCHF from a tertiary-care center [13]; and Pakistan’s most recent State Party Self-Assessment Annual Report (SPAR; 2024) [15] and second Joint External Evaluation (2023) IHR assessments [16].

*Analytical framework:* outbreaks were compared against the IHR 2005 core capacities (surveillance, points of entry [POEs], IPC, risk communication, human resources) and the tripartite One Health framework (integrated human, animal, and environmental surveillance). IPC failures were assessed by cross-referencing outbreak reports with WHO IPC minimum requirements and the IPC Assessment Framework (IPCAF) evaluations conducted in 20 public facilities in Sindh in 2025 [14,16].

*Limitations:* case-level data for the 2024-25 Taunsa outbreak remained partially unpublished at the time of writing; where peer-reviewed data were not yet available, the *Lancet HIV* commentary [8] was triangulated with provincial government statements and named journalistic investigations [9,10]. To the authors’ knowledge, this is the first published analysis linking the 2026 Khairpur neonatal mpox cluster to Pakistan’s broader IPC and IHR failure pattern, based on the search described above. This analysis does not claim outbreak-level statistical comparability; it is a structured qualitative synthesis of health-system failures.

## Findings

### The Khairpur and Karachi clusters

Between January and April 2026, 34 mpox cases were confirmed in Sindh: 26 in Khairpur and Sukkur districts and eight in Karachi [14]; of these, 13 (50%) were children under six months of age, including eight neonates. Among adult cases, 7 of 8 (88%) were female. The index case was identified as a female infant born in February 2026 who developed a rash and was managed across multiple healthcare facilities before dying; she was not tested for mpox, but subsequent confirmed cases among infants admitted to the same facilities suggested an epidemiological link. The WHO joint-mission investigation concluded that the outbreak most likely involved the introduction of mpox virus into healthcare settings through infected neonates, followed by amplification within facilities and household transmission to caregivers, predominantly mothers and female relatives. Multiple public and private healthcare facilities were identified as probable transmission sites [14].

The Karachi cluster (n = 8: six adults, one 13-year-old child, and one 8-day-old infant) included a documented three-case chain in which the primary case had a recent travel history to Central Africa, transmitting to a secondary and then a tertiary case within Karachi—the first evidence of sustained human-to-human clade Ib transmission in Pakistan [14].

### Clade Ib epidemiology and implications for Khairpur

Clade Ib differs from clade IIb in ways that matter for Khairpur. Whereas clade IIb is sustained primarily through sexual networks among men who have sex with men, clade Ib transmits efficiently through household close contact, heterosexual exposure, including sex work, and from mother to infant during pregnancy or delivery, with rapid transmission dynamics and a short incubation period linked to epidemic growth [4,17]. In the eastern DRC, women, sex workers, and children have accounted for a substantial share of morbidity, with a high case-fatality rate in neonates infected vertically or in the first weeks of life. Prospective cohort data from South Kivu reported fetal loss (miscarriage or stillbirth) in half of the pregnant women with confirmed clade I infection [3,4]. Newborn deaths in Khairpur are epidemiologically consistent with this pattern. The transmission story does not require concealed sexual networks; it requires infected pregnant women undiagnosed before delivery, weak IPC in maternity and pediatric facilities, and the absence of a clinical pathway for neonatal mpox. That mechanism sits much closer to the Ratodero, Mirpur Khas, and Taunsa pattern than to anything sexually mediated.

### Cross-cutting IPC and surveillance failures

Despite ratifying the IHR 2005, Pakistan’s core capacities for surveillance, POEs, IPC, and risk communication remain among the weakest in the WHO Eastern Mediterranean Region [15]. Pakistan’s earlier mpox cases, between 2023 and 2025, were imported cases, predominantly clade IIb, arriving through returning travelers from Saudi Arabia and other Gulf states [1,2]. In 2022, subclade IIb caused a global epidemic linked to transmission through sexual contact. The Khairpur cluster is clade Ib, the lineage that drove the August 2024 public health emergency of international concern declaration after rapid expansion in the DRC and onward spread to neighboring countries [18]. The Karachi cluster confirms Central Africa as one importation pathway. POE surveillance configured for Gulf returnees and clade IIb was not designed to detect clade Ib, and syndromic screening is limited by the anatomically concealed nature of many lesions, which evade routine visual assessment.

### One Health: from rhetoric to operational integration

CCHF makes the One Health failure concrete in a way that mpox alone does not. The disease has a livestock reservoir, a tick vector, an annual amplification event in the form of Eid-ul-Azha slaughter, and documented secondary spread in hospital settings. Integrated surveillance across human, veterinary, and environmental systems is what would catch high-risk districts each year before patients reach hospitals, and Pakistan’s integrated One Health surveillance infrastructure remains underdeveloped across the human, veterinary, and environmental sectors. The same district health offices that miss pediatric HIV clusters miss CCHF tick exposure and livestock data and miss imported mpox cases, for the same reasons.

### The IHR 2005 scaffold

Pakistan’s SPAR and the second Joint External Evaluation (2023) flag persistent gaps and limited capacity in POE surveillance, IPC in healthcare facilities, risk communication, and Focal Point coordination with provincial systems [15]. Each gap is visible across all six outbreaks. Imported mpox has entered through major international airports during periods of peak regional transmission; the effectiveness of entry screening is constrained by the limited sensitivity of available tools and the frequency of cutaneous lesions in anatomically concealed sites, which likely contributed to under-detection and under-reporting of imported cases. Pediatric infections from reused syringes signal that injection safety standards exist on paper but not in practice. Nosocomial CCHF transmission to healthcare workers signals that PPE supply and isolation capacity in tertiary hospitals are no better. Risk communication in regional languages remains sparse, leaving rural communities reliant on unregulated practitioners and exposed to uncontrolled livestock contact during the Eid period. These are not new findings. They are the same findings, repeated.

## Priorities

At the provincial level, Sindh requires an immediate IPC audit of all maternity and pediatric facilities in Khairpur and adjacent districts, paired with renewed enforcement against unregulated practitioners. The Ratodero, Mirpur Khas, and Taunsa responses showed that crisis-driven enforcement is possible; sustaining it is the unresolved problem.

At the federal level, the National Institutes of Health, as the IHR Focal Point, should commission an integrated infectious disease surveillance review covering HIV, mpox, CCHF, and injection-related infections, with attention to whether cases are routed through the same district reporting channels and missed at the same junctures. Integrating mpox surveillance into routine sexually transmitted infection services is a key strategy for early detection, in line with the WHO 2024-2026 Global Strategic Preparedness and Response Plan for mpox [19]. Sustainable control requires long-term integration within health systems, strengthened surveillance capacities, and core public health measures to interrupt human-to-human transmission across all settings, consistent with WHO mpox standing recommendations [20]. POE surveillance should be reconfigured for clade Ib pathways through linkage of syndromic surveillance with genomic sequencing and molecular epidemiology, because emerging transmission is unlikely to be confined to traditional Gulf-returnee cohorts. Pre-Eid CCHF surveillance and PPE pre-positioning should be a routine annual activity, not an emergency response. Slaughtering animals on roads or inside houses should be discouraged, and public slaughterhouses should be available in all cities.

Although Pakistan has a national IPC action plan, national IPC guidelines, and cascaded training, the implementation of IPC practices at the facility level remains poor. This requires behavior change in the application of standard precautions for every patient at every encounter, alongside awareness, a dedicated budget, triage and isolation facilities, and trained IPC staff. Administrative support is key to implementation in any institution. Quackery should be banned, syringe reuse should be strictly prohibited, and all healthcare facilities, small or large, should be registered and inspected at regular intervals.

At the policy level, Pakistan needs a clinical pathway for mpox in pregnancy and the neonatal period, in line with current WHO clinical management and IPC guidance [21,22]. This should include antenatal screening protocols in affected districts, IPC standards for delivery and postnatal care of suspected cases, neonatal isolation capacity, procurement of Modified Vaccinia Ankara - Bavarian Nordic for Mpox vaccine for healthcare workers in clade Ib-affected districts, and risk communication in Sindhi, Saraiki, and other regional languages.

Specific antiviral therapy for mpox is not currently registered or stockpiled in Pakistan. Tecovirimat, the only agent with dedicated mpox evidence, is not available through the national essential medicines list or the National Institutes of Health emergency stockpile; brincidofovir and cidofovir are similarly unavailable, and access through WHO and bilateral routes remains slow. Clinical management is therefore limited to supportive and symptomatic care, which is inadequate for severe pediatric and pregnancy-associated disease. The safety and efficacy of these antivirals in neonates are not well established, and adverse effects may pose additional challenges; nonetheless, Pakistan should establish an emergency access pathway and consider smallpox vaccine stockpiling for future prevention.

Neonates and children may differ from adults in terms of complications. Systematic follow-up of infected infants is required to characterize severity, complications, and outcomes. Prevention depends on a high index of suspicion, early detection through Reverse Transcription PCR, prompt isolation of infected mothers, adults, and neonates, appropriate PPE use (gloves, gowns, and masks), and repeated hand hygiene after contact with skin lesions or body fluids. Awareness and education among patients and healthcare workers are the key to ending outbreaks.

## Conclusion

A Pakistani newborn dying of mpox in 2026 is not principally a story about a new virus. The system that allowed HIV outbreaks at Ratodero in 2019, Mirpur Khas in 2024, Taunsa in 2024-2025, and recurrent nosocomial CCHF outbreaks each Eid-ul-Azha has not been rebuilt, and a different pathogen is now exploiting the same gaps. The IHR provides the framework for addressing them. The question is whether Pakistan treats it as a compliance exercise or as the operational standard it was written to be.

## Funding

This research did not receive any specific grant from funding agencies in the public, commercial, or not-for-profit sectors.

## Ethical approval

Not required. This analysis draws on publicly available outbreak reports, surveillance data, and peer-reviewed literature. It does not involve primary research on human participants or identifiable patient data.

## CRediT authorship contribution statement

**Mariyam Sarfraz:** Writing – review & editing, Writing – original draft, Data curation, Conceptualization. **Nadia Noreen:** Writing – review & editing, Data curation. **Nasim Akhtar:** Writing – review & editing.

## Declaration of competing interest

The authors have no competing interests to declare.
